# Multilevel Analysis of Severe Anthropometric Failure among Under-Five Children in Benin: A Cross-Sectional Study Using 2017 to 2018 Demographic and Health Survey Data

**DOI:** 10.5334/aogh.5256

**Published:** 2026-05-13

**Authors:** Estibel Dagne Mekonnen, Nigussie Adam Birhan, Denekew Bitew Belay

**Affiliations:** 1 Laboratory of Biomathematics and Forest Estimations, University of Abomey-Calavi, Cotonou, Benin; 2 Department of Statistics, College of Natural and Computational Science, Injibara University, Injibara, Ethiopia; 3 Department of Statistics, College of Science, Bahir Dar University, Bahir Dar, Ethiopia; 4 School of Health Systems and Public Health, Faculty of Health Sciences, University of Pretoria, Pretoria, South Africa

**Keywords:** composite index of severe anthropometric failure, under-five children, Benin

## Abstract

*Background:* Child undernutrition remains a major global health challenge and a leading cause of child mortality in low- and middle-income countries. To our knowledge, the combined burden of severe malnutrition has not been assessed in Benin. This study aimed to assess the prevalence of severe anthropometric failure and identify associated risk factors among children under five in Benin.

*Methods:* A cross-sectional study was conducted using data from the BDHS 2017–18, including a weighted sample of 11,568 children under five years old. The Composite Index of Severe Anthropometric Failure (CISAF) was used to classify children based on severe stunting, wasting, and underweight. Multilevel logistic regression was employed to identify factors associated with CISAF.

*Results:* The prevalence of CISAF was 12.02%. Factors significantly linked to CISAF included children aged 12–23 months (AOR = 1.62), multiple births (AOR = 3.60), small birth size (AOR = 2.11), having diarrhea (AOR = 1.25), lack of improved water sources (AOR = 1.21), having healthcare access problems (AOR = 1.16), rich wealth index (AOR = 0.52), female children (AOR = 0.66), institutional delivery (AOR = 0.61), having media exposure (AOR = 0.83), urban residents (AOR = 0.85), and high community literacy level (AOR = 0.79).

*Conclusions:* This study found that 12.02% of children under five experienced CISAF. To reduce this burden, stakeholders should expand access to maternal and child healthcare, improve access to safe water, strengthen poverty alleviation programs, and enhance community education and media-based health awareness.

## Introduction

Child undernutrition remains among the most critical of global health concerns and a leading cause of death in low- and middle-income countries [1, 2]. According to the World Health Organization, malnutrition accounts for nearly 45% of all deaths among children under five years of age globally [3]. It is a major public health problem with serious consequences for child survival, growth, cognitive and psychosocial development, and later economic productivity at both individual and societal levels [4–8].

Globally, in 2022, 149 million children under five were stunted, 45 million wasted, and 37 million overweight [9]. Sub-Saharan Africa bears the highest burden of undernutrition among all regions, driven by factors such as poverty, food insecurity, political instability, climate variability, and weaknesses in healthcare systems, which exacerbate children’s nutritional deficiencies [10–12].

Benin, a country in West Africa characterized by high birth rates, low-income levels, and limited access to healthcare, continues to experience a significant rate of child undernutrition. Approximately 36.5% of children under the age of five are affected by chronic malnutrition, a significant public health issue that has the potential to impede cognitive development, immune system function, and overall productivity throughout their lives. This issue reflects persistent challenges related to poverty, food insecurity, and limited access to nutrition services. Despite the implementation of comprehensive measures, such as micronutrient supplementation and community nutrition initiatives, the decline in malnutrition rates remains slow [13].

Undernourishment is not only reflected in stunting (short-for-age), wasting (thinness-for-height), and underweight (thinness-for-age) but also in more severe and combined anthropometric failures. A combined measure of these failures is needed to capture the full extent of child malnutrition [14].

Recent studies increasingly support the use of a more comprehensive measure of child undernutrition known as the Composite Index of Severe Anthropometric Failure (CISAF), introduced by Svedberg [15]. This index combines severe stunting, wasting, and underweight into one exclusive category. It addresses the shortcomings of traditional metrics and provides a more complete assessment than single measures alone. This enhances both the detection of and understanding of the malnutrition burden in populations. Hence, CISAF provides a holistic perspective on anthropometric failure, avoiding the underestimation that can occur when indicators are analyzed in isolation. It has gained traction in global research contexts for its ability to capture the complete extent of undernutrition [16]. Despite this, previous studies in Benin have not incorporated CISAF into child malnutrition assessment, indicating a significant gap in the country’s evidence base. Moreover, earlier studies have often relied on traditional statistical methods, particularly simple logistic regression models, which do not account for the hierarchical structure of health data, where children are nested within households and communities. This oversight can lead to the underestimation of standard errors, potentially by as much as 30%, resulting in misleading conclusions [17]. In contrast, multilevel logistic regression modeling facilitates the partitioning of variance across different levels, namely individual and community, thereby enabling a more comprehensive examination of both contextual and individual risk factors associated with severe anthropometric failure. This methodological approach has been effectively employed in various contexts to identify unmeasured contextual determinants that may influence child health outcomes, such as maternal education level and health service utilization [18].

Previous research in Benin has mostly focused on child malnutrition using individual indicators such as stunting and wasting [19–21]. However, no prior studies have systematically quantified severe anthropometric failure using the CISAF framework. This study aims to estimate the prevalence of severe anthropometric failure among children under five in Benin using CISAF and to examine its individual- and community-level determinants through multilevel logistic regression. The findings are expected to inform context-specific, evidence-based nutrition interventions and policies tailored to Benin and similar low-resource settings.

## Methods

### Study design and setting

This study employed a cross-sectional design to analyze severe anthropometric failures among children under five in Benin. Benin is a West African country covering approximately 114,763 square kilometers and is divided into 12 departments and 77 communes. It is bordered by Togo to the west, Nigeria to the east, Burkina Faso and Niger to the north, and the Atlantic Ocean to the south. The country exhibits diverse geographic features, including coastal plains and plateaus, and a tropical climate that influences agricultural production and food security.

### Data sources and study population

The data for this study were obtained from the most recent Benin Demographic and Health Survey (BDHS) 2017–18, which provides detailed information on health, nutrition, and demographic indicators across all regions of Benin. The BDHS is a collaborative effort led by the National Institute of Statistics and Economic Analysis and the Ministry of Health of Benin, with technical and financial support from international organizations. This includes the United States Agency for International Development and UNICEF. The survey provides critical data to policymakers and stakeholders, facilitating evidence-based interventions to improve child health and nutrition outcomes [22].

### Sample size and sampling procedures

This study analyzed secondary data from the BDHS 2017–18, which included a weighted sample of 11,568 children under five years of age. The BDHS employed a stratified, two-stage cluster sampling design. Stratification was based on the combination of the 12 departments and urban–rural residence, resulting in 24 sampling strata (except Littoral, which has no rural stratum). In the first stage, 555 primary sampling units (PSUs) or clusters were selected from the list of enumeration areas (EAs) established during the fourth General Population and Housing Census conducted in 2013. EAs were selected systematically using probability proportional to size (PPS), with size defined as the number of households in each EA. In the second stage, a household listing was conducted in each selected EA, and 26 households per EA were systematically selected with equal probability. All children aged 0–59 months living in the selected households were included in the survey. Sampling weights were applied to adjust for unequal probabilities of selection and non-response, ensuring nationally representative estimates.

### Variables of the study

In this study, the dependent variable was the CISAF among children aged 0–59 months, coded as 1 = severe anthropometric failure and 0 = no severe failure. The nutritional status of the children was categorized into seven groups, as depicted in Table 1. The calculation of CISAF follows procedures similar to those used for the Composite Index of Anthropometric Failure (CIAF), as described in [23]. Unlike CIAF, which captures a broader spectrum of anthropometric failures by combining stunting, wasting, and underweight into a single index, CISAF focuses exclusively on severe cases of anthropometric failure. Children were classified as severely stunted, wasted, or underweight if their height-for-age, weight-for-height, or weight-for-age *z*-scores were below −3 standard deviations (HAZ < −3, WHZ < −3, WAZ < −3) according to the WHO child growth standards. The severe nutritional indicators for children under five were categorized into eight groups: “no severe failure,” “severe underweight only,” “severe wasting and severe underweight,” “severe wasting and severe stunting,” “severe stunting only,” “severe stunting and severe underweight,” “severe wasting only,” “severe wasting and severe stunting,” and “severe underweight.”

**Table 1:** Classification of the Composite Index of Severe Anthropometric Failure.

GROUP	CISAF	DESCRIPTION	STUNTING	WASTING	UNDERWEIGHT
**A**	No severe failure	Normal WAZ, HAZ, and WHZ	No	No	No
**B**	Severe wasting only	WHZ < −3SD, but normal HAZ and WAZ	No	Yes	No
**C**	Severe wasting and underweight	WAZ and WHZ < −3SD, but HAZ normal	No	Yes	Yes
**D**	Severe stunting, wasting, and underweight	WAZ, WHZ, and HAZ < −3SD	Yes	Yes	Yes
**E**	Severe stunting and underweight	HAZ and WAZ < −3SD, but normal WHZ	Yes	No	Yes
**F**	Severe stunting only	HAZ < −3SD, but normal WAZ and WHZ	Yes	No	No
**G**	Severe underweight only	WAZ < −3SD, but normal HAZ and WHZ	No	No	Yes
**H**	Severe stunting and wasting	HAZ and WHZ < −3SD, but normal WAZ	Yes	Yes	No

Children who did not exhibit any signs of anthropometric failure across categories B–H were classified as “no severe failure,” indicating normal, mild, or moderate undernutrition. According to the CISAF standard, a child was classified as having “severe anthropometric failure” if they exhibited any of the conditions listed in categories B–H, as shown in Table 1.

This study examined several independent variables that influence the CIAF, selected based on a review of relevant literature [24, 25] and data availability in the BHDS dataset. The explanatory variables include both individual- and community-level factors. Individual-level variables include: household wealth index, mother’s age at first birth, maternal education, maternal occupation, sex of household head, marital status, ANC level, place of delivery, mother’s breastfeeding status (during survey time), number of children under five, healthcare access, media exposure, sex of child, child age, birth size, birth type, birth order, vitamin A supplement, and diarrhea. Community-level variables like poverty, literacy, and media exposure were derived by aggregating individual responses within each survey cluster. Clusters were classified as low or high based on the median proportion of poor households, educated mothers, or individuals exposed to media, capturing neighborhood-level influences on children’s anthropometric outcomes [26].

### Data processing and analysis

Data cleaning and analysis were conducted using STATA/SE version 18.0. Sampling weights, clustering, and stratification were accounted for to obtain nationally representative estimates. Some continuous variables were categorized using standard cut-offs. Descriptive statistics, including frequencies and percentages, were used to summarize the study population and the prevalence of severe anthropometric failure.

Multilevel logistic regression models were fitted to examine the associations between predictor variables and the odds of severe anthropometric failure, accounting for the hierarchical structure of the data (children nested within households and communities). Four models were constructed: a null model (without predictors), an individual-level model, a community-level model, and a combined model of individual and community variables. Model fit was assessed using the Akaike Information Criterion (AIC) and log-likelihood ratio tests, with lower AIC values indicating better fit.

Fixed effects were used to estimate the association between the likelihood of CISAF and explanatory variables. The result was expressed as an adjusted odds ratio with a 95% confidence interval. Random effects were quantified using the intra-class correlation coefficient (ICC), median odds ratio, and proportional change in variance (PCV) to estimate the extent of variation attributable to clustering at community and household levels. Adjusted odds ratios (AORs) with 95% confidence intervals (CIs) were reported, with a significance level set at *p* < 0.05.

## Results

### Descriptive analysis

The study analyzed a weighted sample of 11,568 children under the age of five. Table 2 presents their characteristics based on CISAF in Benin. About 6854 (59.25%) of the participants resided in rural areas, and 2467 (21.33%) participants were from the poorest wealth index family. Most of the respondents, 10,923 (94.42%), were married or cohabiting with a partner; 1947 (16.83%) had attained secondary education or higher, and 7511 (64.93%) had not received any formal education.

**Table 2:** Characteristics of children under five by CISAF in Benin (*N* = 11,568).

VARIABLE	CATEGORIES	CISAF		TOTAL
		NO SEVERE FAILURE *N* (%)	SEVERE FAILURE *N* (%)	
**Sex of child**	Male	5040 (86.3%)	802 (13.7%)	5842
	Female	5138 (89.7%)	588 (10.3%)	5726
**Child age (months)**	0–11	2571 (91.6%)	236 (8.4%)	2807
	12–23	2097 (87.5%)	299 (12.5%)	2396
	24–35	1815 (84.6%)	331 (15.4%)	2146
	36–47	1877 (86.3%)	298 (13.7%)	2175
	48–59	1818 (88.9%)	226 (11.1%)	2044
**Wealth quintile**	Poorest	2048 (83.0%)	419 (17.0%)	2467
	Poorer	1934 (84.7%)	350 (15.3%)	2284
	Middle	2043 (88.6%)	264 (11.4%)	2307
	Richer	2059 (89.7%)	236 (10.3%)	2295
	Richest	2094 (94.5%)	121 (5.5%)	2215
**Sex of household head**	Male	8555 (88.0%)	1169 (12.0%)	9724
	Female	1623 (88.0%)	221 (12.0%)	1844
**Marital status**	Married/living with partner	9617 (88.0%)	1306 (12.0%)	10,923
	Other	561 (87.0%)	84 (13.0%)	645
**Toilet type**	Improved	3007 (92.2%)	256 (7.8%)	3263
	Unimproved	7171 (86.3%)	1134 (13.7%)	8305
**Source of water**	Improved	5698 (89.3%)	682 (10.7%)	6380
	Unimproved	4480 (86.3%)	708 (13.7%)	5188
**Electricity access**	Yes	3482 (92.1%)	299 (7.9%)	3781
	No	6696 (86.0%)	1091 (14.0%)	7787
**Mobile phone access**	Yes	5250 (91.2%	504 (8.8%)	5754
	No	4928 (84.8%)	886 (15.2%)	5814
**Type of residence**	Urban	4280 (90.8%)	434 (9.2%)	4714
	Rural	5898 (86.1%)	956 (13.9%)	6854
**Region**	Alibori	1166 (83.4%)	232 (16.6%)	1398
	Atacora	960 (84.7%)	174 (15.3%)	1134
	Atlantic	974 (88.9%)	122 (11.1%)	1096
	Borgou	1209 (86.0%)	197 (14.0%)	1406
	Collines	865 (93.3%)	62 (6.7%)	927
	Couffo	664 (87.8%)	92 (12.2%)	756
	Donga	749 (88.9%)	94 (11.1%)	843
	Littoral	709 (93.2%)	52 (6.8%)	761
	Mono	517 (88.5%)	67 (11.5%)	584
	Ouémé	812 (90.3%)	87 (9.7%)	899
	Plateau	589 (85.6%)	99 (14.4%)	688
	Zou	964 (89.6%)	112 (10.4%)	1076
**Number of children under five**	1	2483 (90.0%)	277 (10.0%)	2760
	2	4193 (89.2%)	509 (10.8%)	4702
	≥3	3502 (85.3%)	604(14.7%)	4106
**Vitamin A supplement**	Yes	5241 (88.9%)	654 (11.1%)	5895
	No	4937 (87.0%)	736 (13.0%)	5673
**Recent diarrhea**	Yes	1097 (84.9%)	195 (15.1%)	1292
	No	9081 (88.4%)	1195 (11.6%)	10,276
**Type of birth**	Single	9836 (88.8%)	1246 (11.2%)	11,082
	Multiple	342 (70.4%)	144 (29.6%)	486
**Birth order**	First	2162 (88.7%)	276 (11.3%)	2438
	2nd to 3rd	3737 (88.6%)	481 (11.4%)	4218
	4th to 5th	2421 (87.8%)	337 (12.2%)	2758
	≥6th	1858 (86.3%)	296 (13.7%)	2154
**Place of delivery**	Home	1368 (78.7%)	370 (21.3%)	1738
	Health institution	8810 (89.6%)	1020 (10.4%)	9830
**Mother’s education**	No education	6475 (86.2%)	1036 (13.8%)	7511
	Primary	1889 (89.5%)	221 (10.5)	2110
	Secondary and above	1814 (93.2%)	133 (6.8%)	1947
**Mother’s occupation**	Has work	8499 (88.1%)	1144 (11.9%)	9643
	No work	1679 (87.2)	246 (12.8%)	1925
**Mother’s breastfeeding status (during survey time)**	Yes	6227 (88.3%)	825 (11.7%)	7052
	No	3951 (87.5%)	565 (12.5%)	4516
**Healthcare access**	Not a big problem	4123 (89.6%)	477 (10.4%)	4600
	Big problem	6055 (86.9%)	913 (13.1%)	6968
**Mother’s age at first birth**	≤17	2614 (85.3%)	452 (14.7%)	3066
	18–24	6436 (88.9%)	808 (11.1%)	7244
	≥25	1128 (89.7%)	130 (10.3%)	1258
**Birth size**	Large	2926 (90.7%)	301 (9.3%)	3227
	Average	5830 (88.6%)	752 (11.4%)	6582
	Small	1422 (80.8%)	337 (19.2%)	1759
**ANC level**	Not adequate	6393 (86.2%)	1020 (13.8%)	7413
	Adequate	3785 (91.1%)	370 (8.9%)	4155
**Media exposure**	Yes	6358 (89.9%)	714 (10.1%)	7072
	No	3820 (85.0%)	676 (15.0%)	4496
**Community media exposure**	Low	4971 (85.7%)	832 (14.3%)	5803
	High	5207 (90.3%)	558 (9.7%)	5765
**Community literacy level**	Low	4920 (84.8%)	882 (15.2%)	5802
	High	5258 (91.2%)	508 (8.8%)	5766
**Community poverty level**	Low	5296 (91.1%)	516 (8.9%)	5812
	High	4882 (84.8%)	874 (15.2%)	5756

*Note:* Percentages are calculated within each category (row percentages).

**Figure 1:**
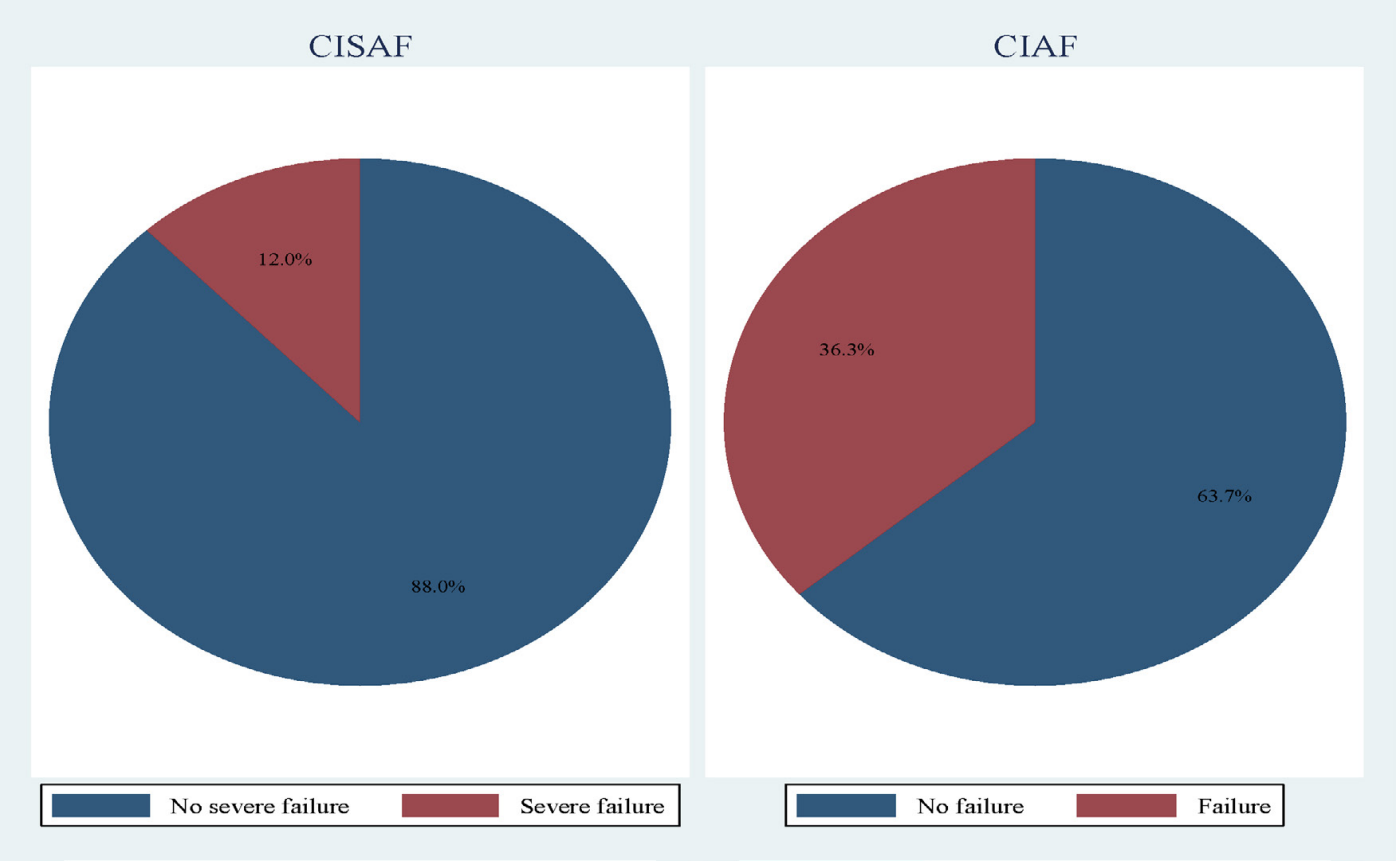
Proportion of CISAF and CIAF among under-five children in Benin.

Figure 1 compares CISAF and CIAF in children under five in Benin, showing that 12.02% had severe anthropometric failure, and 36.3% had one form of failure based on CIAF.

About 7.78% of the children were severely stunted only, which points to the chronic nature of undernutrition in Benin. Severe stunting is therefore the most prevalent form of anthropometric failure, suggesting its enduring impact on child growth and development (Table 3).

**Table 3:** Prevalence of CISAF and its subgroups among children under five in Benin (*N* = 11,568).

GROUP	CISAF	STUNTING	WASTING	UNDERWEIGHT	(%)
**A**	No severe failure	No	No	No	87.98
**B**	Severe wasting only	No	Yes	No	0.48
**C**	Severe wasting and underweight	No	Yes	Yes	0.37
**D**	Severe stunting, wasting, and underweight	Yes	Yes	Yes	0.24
**E**	Severe stunting and underweight	Yes	No	Yes	2.52
**F**	Severe stunting only	Yes	No	No	7.78
**G**	Severe underweight only	No	No	Yes	0.00
**H**	Severe stunting and wasting	Yes	Yes	No	0.62
					**100**
**CISAF**		**B+C+D+E+F+G+H**			**12.02%**

### Multilevel binary logistic regression analysis

The ICC from the null model was 0.34 (95% CI: 0.25, 0.47), indicating that approximately 34% of the variation in CISAF was attributable to differences between clusters (enumeration areas). The MOR value of 1.97 suggests moderate clustering of severe anthropometric failure among children under five. These results indicate substantial between-cluster variability in CISAF. The model with the lowest deviance and high log-likelihood was selected as the best-fitted model; thus, model IV was retained (Table 4).

**Table 4:** Random effects and model fit statistics for predictors of CISAF among children under five in Benin.

PARAMETER	NULL (MODEL I)	MODEL II	MODEL III	MODEL IV
**Community-level variance**	0.3437	0.1712	0.1883	0.1345
**ICC**	0.0946	0.0494	0.0541	0.03928
**PCV**	reference	50.1803	45.1814	60.8646
**MOR**	1.9703	1.6069	1.6446	1.5523
**AIC**	8396.15	7887.34	8300.11	7885.58
**Model fitness**				
**Log-likelihood**	−4198.07	−3914.67	−4144.05	−3898.79
**Deviance (−2LL)**	8396.14	7829.34	8288.10	7797.58

Table 5 presents the results of the multilevel logistic regression model. Individual-level characteristics include child’s sex, age, birth type, and birth size, as well as maternal factors such as age at birth, media exposure, and wealth index. Community-level factors, including community literacy level and place of residence, were significantly associated with CISAF among children under five in Benin.

**Table 5:** Predictors of the severe composite index of anthropometric failure among children under five in Benin.

VARIABLES	MODEL II AOR (95% OF CI)	MODEL III AOR (95% OF CI)	MODEL IV AOR (95% OF CI)
**Sex of child**			
**Male**	Ref		Ref
**Female**	0.66 (0.59, 0.75)*		0.66 (0.59, 0.75)*
**Age of child (months)**			
**0–11**	Ref		Ref
**12–23**	1.64 (1.35, 1.98)*		1.62 (1.34, 1.96)*
**24–35**	2.21 (1.83, 2.66)*		2.20 (1.83, 2.65)*
**36–47**	1.86 (1.53, 2.26)*		1.85 (1.53, 2.25)*
**48–59**	1.40 (1.13, 1.71)*		1.39 (1.13, 1.70)*
**Wealth quintile**			
**Poorest**	Ref		Ref
**Poorer**	1.07 (0.90, 1.27)		1.07 (0.90, 1.28)
**Middle**	0.81 (0.66, 0.98)*		0.82 (0.67, 1.00)
**Richer**	0.81 (0.64. 1.01)		0.84 (0.66, 1.09)
**Richest**	0.49 (0.35, 0.67)*		0.52 (0.36, 0.74)*
**Source of water**			
**Improved**	Ref		Ref
**Unimproved**	1.24 (1.09, 1.40)*		1.21 (1.06, 1.37)*
**Electricity**			
**No**	Ref		Ref
**Yes**	1.02 (0.84, 1.23)		1.04 (0.85, 1.26)
**Access to mobile phone**			
**No**	Ref		Ref
**Yes**	0.82 (0.71, 0.95)*		0.85 (0.73, 0.97)*
**Recent diarrhea prevalence**			
**No**	Ref		Ref
**Yes**	1.26 (1.05, 1.5)*		1.25 (1.05, 1.50)*
**Birth type**			
**Single**	Ref		Ref
**Multiple**	3.57 (2.84, 4.49)*		3.60 (2.86, 4.52)*
**Birth order**			
**First**	Ref		Ref
**2nd–3rd**	0.89 (0.75, 1.05)		0.89 (0.76, 1.06)
**4th–5th**	0.84 (0.70, 1.02)		0.85 (0.71, 1.03)
**6th and above**	0.76 (0.62. 0.93)*		0.77 (0.63, 0.94)*
**Delivery place**			
**Home**	Ref		Ref
**Health institution**	0.59 (0.51, 0.70)*		0.61 (0.51, 0.72)*
**Mother’s education**			
**No education**	Ref		Ref
**Primary**	0.98 (0.83, 1.17)		1.05 (0.88, 1.25)
**Secondary and higher**	0.74 (0.59, 0.92)*		0.80 (0.63, 1.00)
**Healthcare access**			
**Not a big problem**	Ref		Ref
**Big problem**	1.17 (1.03, 1.34)*		1.16 (1.01, 1.33)*
**Mother’s age at first birth**			
**≤17**	Ref		Ref
**18–24**	0.83 (0.72, 0.95)*		0.83 (0.72, 0.95)*
**25+**	0.84 (0.67, 1.06)		0.83 (0.66, 1.04)
**Birth size**			
**Large**	Ref		Ref
**Average**	1.25 (1.08, 1.45)*		1.27 (1.09, 1.48)*
**Small**	2.12 (1.76, 2.54)*		2.11 (1.75, 2.54)*
**ANC level**			
**Not adequate**	Ref		Ref
**Adequate**	0.89 (0.77, 1.03)		0.87 (0.75, 1.01)
**Media exposure**			
**No**	Ref		Ref
**Yes**	0.86 (0.75, 0.98)*		0.83 (0.72, 0.95)*
**Residence**			
**Rural**		Ref	Ref
**Urban**		0.77 (0.66, 0.91)*	0.85 (0.72, 1.00)
**Community literacy level**			
**Low**		Ref	Ref
**High**		0.70 (0.59, 0.83)*	0.79 (0.66, 0.95)*
**Community poverty level**			
**Low**		Ref	Ref
**High**		1.28 (1.07, 1.54)*	0.96 (0.79, 1.17)
**Community media exposure**			
**Low**		Ref	Ref
**High**		0.85 (0.71, 1.00)	1.00 (0.84, 1.20)
**Region**			
**Alibori**		Ref	Ref
**Atacora**		0.95 (0.71, 1.00)	0.91 (0.68, 1.22)
**Atlantic**		1.02 (0.74, 1.40)	1.07 (0.77, 1.49)
**Borgou**		0.88 (0.67, 1.16)	0.82 (0.62, 1.09)
**Collines**		0.46 (0.32, 0.66)*	0.55 (0.38, 0.79)*
**Couffo**		0.93 (0.67, 1.29)	1.04 (0.74, 1.45)
**Donga**		0.78 (0.56, 1.10)	0.80 (0.56, 1.12)
**Littoral**		0.81 (0.54, 1.21)	1.11 (0.72, 1.71)
**Mono**		0.95 (0.66, 1.38)	0.97 (0.66, 1.42)
**Ouémé**		0.85 (0.61, 1.19)	0.95 (0.67, 1.35)
**Plateau**		1.04 (0.75, 1.44)	1.14 (0.81, 1.59)
**Zou**		0.77 (0.56, 1.04)	1.01 (0.74, 1.40)

*Note:* AOR = adjusted odds ratio; CI = confidence interval; *p < 0.05 indicates statistical significance.

The odds of being a CISAF for a female child were significantly lower compared to male counterparts (AOR: 0.66; 95% CI: 0.59–0.75). Children aged 12–23 months were 1.62 times (AOR: 1.62; 95% CI: 1.34–1.96), 24–35 months were 2.20 times (AOR: 2.20; 95% CI: 1.83–2.65), 36–47 months were 1.85 times (AOR: 1.85; 95% CI: 1.53–2.25), and 48–59 months were 1.39 times (AOR: 1.39; 95% CI: 1.13–1.70) more likely to have CISAF compared to the 0–11 months age group. Similarly, children from the richest households had 48% lower odds of CISAF compared with those from the poorest households (AOR: 0.52; 95% CI: 0.36–0.74).

Lack of access to improved water sources was associated with higher odds of severe anthropometric failure (AOR: 1.21; 95% CI: 1.06–1.37). Conversely, mobile phone ownership was protective (AOR: 0.85; 95% CI: 0.73–0.97). Children who had diarrhea (AOR: 1.25; 95% CI: 1.05–1.50), were born as multiples (AOR: 3.60; 95% CI: 2.86–4.52), and had a smaller birth size (AOR: 2.11; 95% CI: 1.75–2.54) were significantly more likely to experience CISAF. Similarly, children delivered in a health facility had lower odds of CISAF compared with those delivered at home (AOR: 0.61; 95% CI: 0.51–0.72). Likewise, mothers aged 18–24 years had lower odds of CISAF compared to those born to mothers aged 17 years or younger (AOR: 0.83; 95% CI: 0.72–0.95).

Children from households where access to healthcare was reported as a big problem had higher odds of CISAF compared with their counterparts (AOR: 1.16; 95% CI: 1.01–1.33). Higher maternal media exposure was protective against CISAF (AOR: 0.83; 95% CI: 0.72–0.95). In the urban–rural context, urban children had lower odds of CISAF compared to their rural counterparts (AOR: 0.85; 95% CI: 0.72–1.00). Moreover, high community literacy was associated with reduced CISAF (AOR: 0.79; 95% CI: 0.66–0.95). Children residing in the Collines region had lower odds of CISAF compared to those in Alibori (AOR: 0.55; 95% CI: 0.38–0.79).

## Discussions

This research has identified that 12.02% of children under five years old in Benin experience undernutrition, as measured by the CISAF. This result is comparable with studies conducted in Ethiopia (13.9%) [27], Nepal (12.6%) [28], and Bangladesh (11.0%) [16]. However, it is lower than a study conducted in Pakistan (19.4%) [28]. The differences in prevalence across countries might be factors such as socioeconomic disparities, access to healthcare, cultural beliefs and practices, and the availability of food [27]. Considering that stunting affects approximately one-third of children under five in Benin [29], this study provides a more comprehensive picture of undernutrition by capturing children with one or more concurrent forms of undernutrition.

Findings from this study show that male children are about 34% less likely to achieve anthropometric success than female children, consistent with earlier studies conducted in Benin [19], Ethiopia [27], and Nigeria [30]. This disparity may be attributed to biological vulnerabilities such as increased susceptibility to infections and growth differences alongside gender-specific caregiving practices [31]. The study also revealed that children in the older age groups had a higher odds of CISAF than those in younger age groups, consistent with a previous study conducted in Bangladesh [16]. This pattern may reflect increased vulnerability during the 12–35-month age window, a critical developmental period characterized by greater exposure to infections and suboptimal nutritional intake.

The findings of this study also show that children being born as multiples significantly elevate the risk of CISAF, with multiples exhibiting more than threefold higher odds compared to singletons. This finding aligns with a previous study conducted in sub-Saharan Africa [32], which suggests that multiple births are associated with a heightened risk of poor nutritional status due to low birth weight, premature birth, and increased competition for maternal resources.

In contrast, children of higher birth order had lower odds of CISAF compared to firstborns. Most studies in low- and middle-income countries, however, report that higher birth order increases the risk of undernutrition. Some evidence suggests that later-born children may perform equally well or even better when mothers gain experience, adequate birth spacing, or higher levels of education [33–36]. Parents may also improve their child care and feeding practices with each successive child, benefiting later-born children [33]. Longer birth intervals can further reduce the usual disadvantages for higher-order children [37].

Household socioeconomic status significantly influences children’s nutritional status. Children from the wealthiest households were less likely to experience CISAF compared with those from the poorest households [16, 23]. Furthermore, an unimproved water source raises the likelihood of being CISAF. The results of this investigation are in line with findings from a study conducted in Ethiopia [38]. Additionally, children born in health facilities had lower odds of CISAF, consistent with evidence that institutional delivery is associated with improved neonatal care and access to child health services [39].

Maternal characteristics significantly influence child nutrition outcomes. Children of mothers with secondary or higher education levels exhibited reduced odds of CISAF. This study is consistent with studies conducted in Bangladesh [16] and Ethiopia [27]. One possible explanation is that formal education equips mothers with the knowledge and skills to adopt appropriate feeding and hygiene practices, seek timely healthcare for childhood illnesses, and engage in behaviors that reduce the risk of undernutrition.

Mothers who began childbearing between the ages of 18 and 24 were less likely to have undernourished children compared to those who began in their teenage years. This is consistent with a previous study conducted in Pakistan [40]. This may be explained by delayed childbearing, which is associated with improved maternal and child health. Children with small birth sizes had higher odds of CISAF, highlighting the long-term effects of intrauterine growth restriction and low birth weight on growth patterns [16, 41].

Media exposure also mitigated the risk of CISAF, demonstrating that mass media can promote optimal child feeding and care practices [16]. Conversely, mothers who perceived access to healthcare as a significant issue had children with higher probabilities of failure, reflecting structural barriers such as distance, affordability, and quality of care that impede the effective utilization of health [42].

Moreover, children residing in urban areas have 0.85 times the odds of experiencing anthropometric failure compared to their rural counterparts. These findings are consistent with studies conducted in Bangladesh [43, 44], which report a higher burden of undernutrition among children under five in rural areas than in urban settings. Additionally, communities with higher literacy levels exhibited lower odds of CISAF, likely reflecting the broader benefits of health knowledge and the adoption of improved childcare practices [45].

Overall, this rigorous analytical approach ensures a nuanced understanding of both individual and community determinants of severe anthropometric failure in Benin, providing evidence to inform targeted nutrition policies and interventions.

## Conclusions

This study found that 12.02% of children under five were affected by CISAF, highlighting undernutrition as a major public health concern in Benin. Multiple individual- and community-level factors were significantly associated with CISAF. Addressing this burden requires expanding access to quality maternal and child healthcare, improving water and sanitation infrastructure, strengthening poverty reduction efforts, and promoting community education and media-based health awareness.

## Figures and Tables

**Table:** 

AIC	Akaike Information Criteria
ANC	Antenatal care
AOR	Adjusted Odds Ratio
BDHS	Benin Demography and Health Survey
BIC	Bayesian Information Criteria
CI	Confidence Interval
CIAF	Composite Index of Anthropometric Failure
CISAF	Composite Index of Severe Anthropometric Failure
EA	Enumeration area
HAZ	Height for age standardized score
MOR	Median odds ratio
PCV	Proportional Change in Variance
SD	Standard deviation
UNICEF	United Nations Population Fund
WAZ	Weight for age standardized score
WHZ	Weight for height standardized score

## Data Availability

The data used in this study are publicly accessible and available from the DHS website (https://dhsprogram.com/data/available-datasets.cfm). The name of the dataset is Benin Demographic and Health Survey (BDHS) 2017–18.
